# Different Thymosin Beta 4 Immunoreactivity in Foetal and Adult Gastrointestinal Tract

**DOI:** 10.1371/journal.pone.0009111

**Published:** 2010-02-09

**Authors:** Sonia Nemolato, Tiziana Cabras, Flaviana Cau, Mattia Umberto Fanari, Daniela Fanni, Barbara Manconi, Irene Messana, Massimo Castagnola, Gavino Faa

**Affiliations:** 1 Divisione di Anatomia Patologica, Dipartimento di Citomorfologia, University of Cagliari, Cagliari, Italy; 2 Dipartimento di Scienze Applicate ai Biosistemi, Università di Cagliari, Cagliari, Italy; 3 Istituto di Biochimica e di Biochimica Clinica, Università Cattolica and/or Istituto per la Chimica del Riconoscimento Molecolare, CNR, Istituto Scientifico, Internazionale (ISI) Paolo VI, Roma, Italy; The University of Hong Kong, China

## Abstract

**Background:**

Thymosin beta 4 (Tβ_4_) is a member of beta-thymosins, a family of peptides that play essential roles in many cellular functions. A recent study from our group suggested a role for Tβ_4_ in the development of human salivary glands. The aim of this study was to analyze the expression of Tβ_4_ in the human gut during development, and in the adult.

**Methodology/Principal Findings:**

Immunolocalization of Tβ_4_ was studied in autoptic samples of tongue, oesophagus, stomach, ileum, colon, liver and pancreas obtained from two human foetuses and two adults. Tβ_4_ appeared unevenly distributed, with marked differences between foetuses and adults. In the stomach, superficial epithelium was positive in foetuses and negative in adults. Ileal enterocytes were strongly positive in the adult and weakly positive in the foetuses. An increase in reactivity for Tβ_4_ was observed in superficial colon epithelium of adults as compared with the foetuses. Striking differences were found between foetal and adult liver: the former showed a very low reactivity for Tβ_4_ while in the adult we observed a strong reactivity in the vast majority of the hepatocytes. A peculiar pattern was found in the pancreas, with the strongest reactivity observed in foetal and adult islet cells.

**Significance:**

Our data show a strong expression of Tβ_4_ in the human gut and in endocrine pancreas during development. The observed differential expression of Tβ_4_ suggests specific roles of the peptide in the gut of foetuses and adults. The observed heterogeneity of Tβ_4_ expression in the foetal life, ranging from a very rare detection in liver cells up to a diffuse reactivity in endocrine pancreas, should be taken into account when the role of Tβ_4_ in the development of human embryo is assessed. Future studies are needed to shed light on the link between Tβ_4_ and organogenesis.

## Introduction

Beta-thymosins (Tβs) constitute a highly conserved family of actin-binding polypeptides [1], presenting a well conserved four-aminoacid motif, corresponding to the sequence LKKT, which interacts with actin, promoting or inhibiting actin assembly [2]. Thymosin beta-4 (Tβ_4_) is the archetypal member of the beta-thymosins family: it is a 43-aminoacid peptide, isolated from human blood platelets [3] which forms a 1∶1 complex with actin, inhibits its polymerization [4], and acts as an extremely effective actin-monomer sequestering peptide [5]. Tβ_4_ has multiple functions: it moonlights to repair injured tissues [6], has anti-inflammatory efficacy in monocyte/macrophages [7], promotes wound healing [8] and mediates angiogenesis [9]. Tβ_4_ has been also shown to play a relevant role during the development of different neural cell types in the rat brain [10]. In particular, Tβ_4_ plays a neurotrophic and antiapoptotic role during the development of the nervous system [11]. In the embryo, reduced myocardial Tβ_4_ levels have been reported to cause a disrupted coronary vasculogenesis and a number of cardiac defects [12]. During coronary vessels development, Tβ_4_ should act on cardiac stem cells, also known as epicardially derived cells (EPDCs) [13] inducing their migration into the myocardium, where they differentiate into either endothelial or smooth muscle cells [9]. The theory on the putative role of Tβ_4_ in the physiological development of embryos, as well as in vascularization and tissue recovery in acute and chronic ischemia, has been reinforced by the discovery that Tβ_4_ is one of the most abundant factors secreted by embryonic endothelial progenitor cells [14]. Recently a study from our group evidenced a strong reactivity for Tβ_4_ in developing foetal salivary glands, with a switch from the acinar component to the ductal cells in the adult [15]. Tβ_4_ has also been identified as a predominant transcript in intraepithelial lymphocytes (IEL) in the murine gut [16], in which it could exert a relevant anti-inflammatory effect by inhibiting neutrophilic infiltration [17].

On the basis of these data, suggesting a relevant role for Tβ_4_ during the development of the foetus and the embryo, it seemed of some interest to investigate the expression of the peptide in the gastrointestinal tract of human fetuses and in adults, with the aim to gain insights into the expression of Tβ_4_ in the human gastrointestinal tract during development.

## Materials and Methods

**Figure 1:**
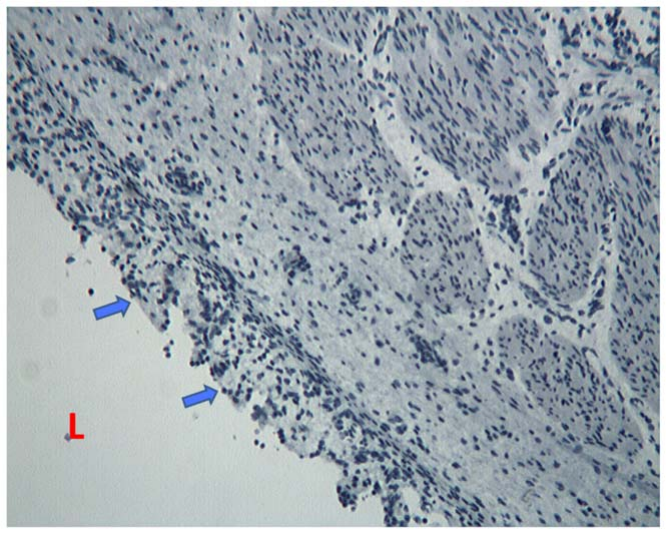
Negative control. Foetal oesophagus immunostained by using isotype antibody. L =  oesophageal lumen. Arrows indicate the surface epithelium.

Two human fetuses of 20 weeks (male) and 21 weeks (female) of gestational age, and two adults 65 and 74 year old respectively were the object of the present study. In each subject, we obtained, at autopsy, multiple samples from the following segments of the gut: tongue, oesophagus, stomach, ileum, colon. Samples were also obtained from liver and pancreas. Tissue samples were fixed in 10% formalin, routinely processed and paraffin-embedded. Immunohistochemistry was performed on 5 µm-thick sections, using the labeled streptavidin-biotin complex system (LSAB2, Dako) in a Dako Autostainer (DakoCytomation, Carpintera, CA, USA). Heat-induced antigen retrieval was carried out by steaming unstained sections in Target Retrieval Solution (Dako TRS pH 6.1) for 30 min. Tissue sections were incubated (30 min at room temperature) with the monoclonal anti-thymosin beta 4 antibody (Bachem, Bubendorf, Switzerland). Sections of a reactive human adult lymph node with activated macrophages were used as positive controls. As a negative control, we utilized sections of foetal oesophagus: immunoistochemistry was performed using isotype antibody (Fig. 1). The immunostaining was interpreted as positive when at least 10% of cells expressed the antigen. The positive expression was further categorized into focal (10–33%) and diffuse (>33%).

All cases were independently reanalyzed by two pathologists specialized in gastrointestinal pathology (GF, SN).

### Ethics Statements

The study protocol and written consent forms were approved by the Ethics Human Studies Committee of University Medical Centre of Cagliari (according to the instructions of the declaration of Helsinki). Full written consent forms were obtained from the parents of the newborns and all rules were respected. For the specimens from adults, we obtained written consent to revearch use by their next of kin.

## Results

The immunostaining for Tβ_4_ appeared granular or homogeneously diffuse, always restricted to the cytoplasm of positive cells; no nuclear reactivity was observed in this study. Reactivity for the peptide was also observed, in some cases, in the interstitial spaces, as fine granules, mainly located around the epithelial structures. No significant differences were found in the immunoistochemical pattern for Tβ_4_ between the two foetuses and the two adults analyzed.

### Tongue

#### Foetus (20 weeks of gestation)

**Figure 2:**
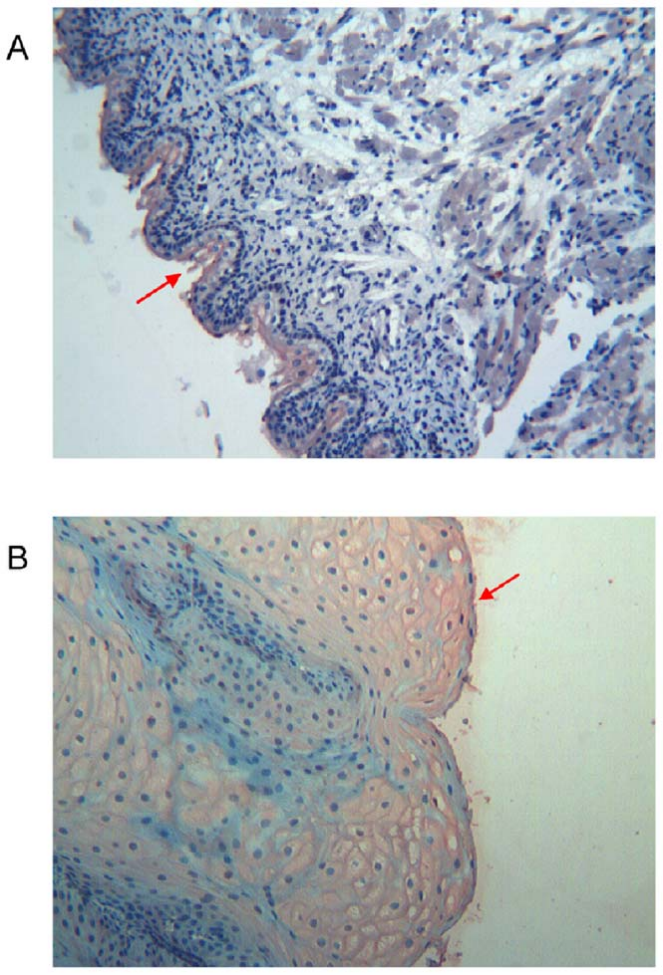
Immunohistochemical detection of thymosin β4 in foetal and adult tongue. **a**) Foetal tongue: the superficial epithelial cells (arrow) shows a diffuse immunoreactivity for Tβ_4_, localized in the cytoplasm. The underlying corion is negative. (Original Magnification ×250) **b**) Adult tongue: the superficial layers of the stratified epithelium show a strong diffuse cytoplasmic reactivity for Tβ_4_ (arrow). (Original Magnification ×250)

The epithelium covering the tongue appeared constantly negative in its deeper layers. The superficial epithelial cells showed a diffuse immunoreactivity for Tβ_4_, localized in the cytoplasm (Fig. 2a). The underlying corion was negative. A weak positivity was also observed in the muscular cells.

#### Adult

The immunoistochemical pattern paralleled that detected in the foetuses. The superficial layers of the stratified epithelium of the tongue showed a diffuse cytoplasmic reactivity for Tβ_4_ (Fig. 2b).

### Oesophagus

#### Foetus (21 weeks of gestation)

**Figure 3:**
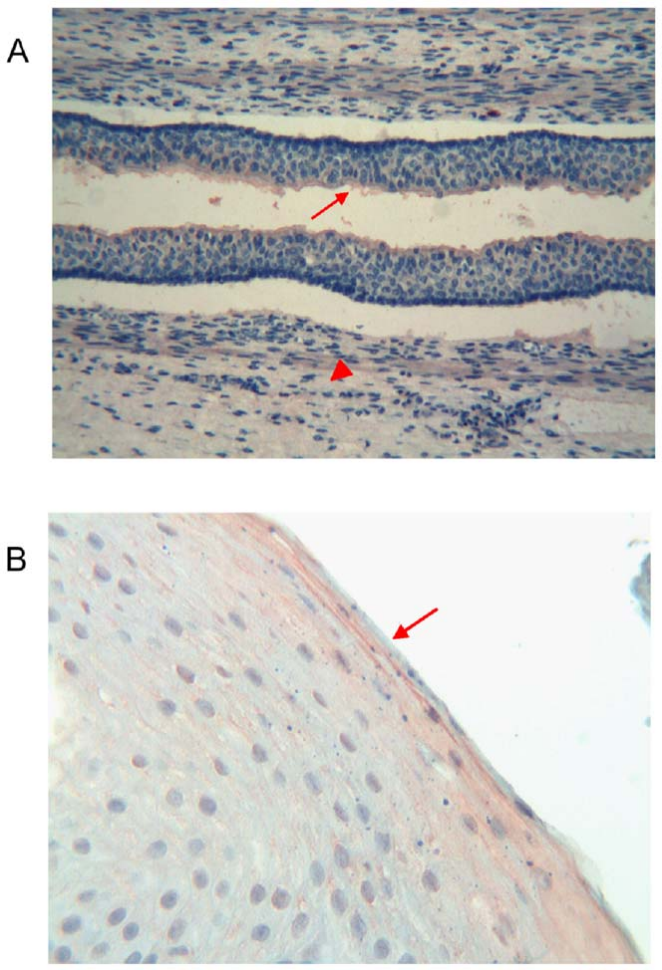
Immunohistochemical detection of thymosin β4 in foetal and adult oesophagus. **a**) Foetal oesophagus: the oesophageal lumen is coated with a thin Tβ_4_-immunoreactive layer (arrow). A weak reactivity for the peptide is observed in the muscular cells (arrowhead). (Original Magnification ×400) **b**) Adult oesophagus: immunoreactivity for Tβ_4_ is restricted to epithelial cells of the superficial layers (arrow). (Original Magnification ×400)

The epithelium covering the oesophageal lumen appeared negative. The oesophageal lumen appeared coated with a thin Tβ_4_-immunoreactive layer, which resulted diffuse to the entire oesophageal lumen (Fig. 3a). A weak reactivity for the peptide was observed in the muscular cells.

#### Adult

Immunoreactivity for Tβ_4_ was restricted to epithelial cells of the superficial layers covering the esophageal lumen. No reactivity for Tβ_4_ was found in the intermediated and deep layers (Fig. 3b).

### Stomach

#### Foetus (21 weeks of gestation)

**Figure 4:**
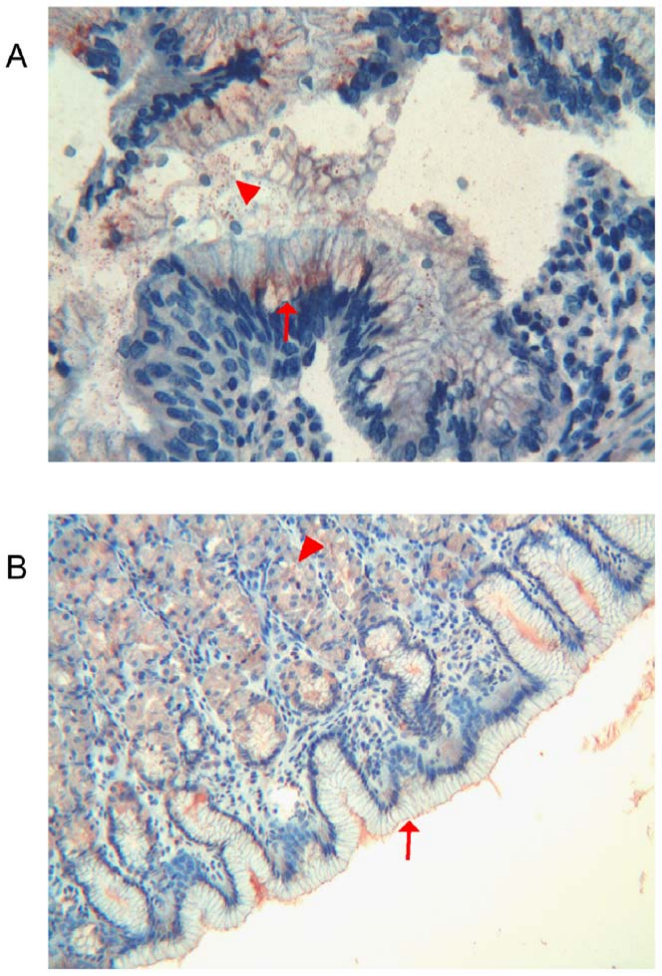
Immunohistochemical detection of thymosin β4 in foetal and adult stomach. **a**) Foetal stomach: Tβ_4_-immunoreactive granules are shown in the cytoplasm of the majority of columnar cells (arrow). Tβ_4_ granular deposits are also found in mucous covering the gastric surface (arrowhead). (Original Magnification ×400) **b**) Adult stomach: gastric mucosa appears covered by a thin superficial layer intensely reactive for Tβ_4_ (arrow). Immunoreactivity for Tβ_4_ in gastric glands is restricted to chief and oxyntic cells (arrowhaed). (Original Magnification ×250)

The gastric epithelium showed the presence of Tβ_4_-immunoreactive granules in the cytoplasm of the majority of columnar cells. Immunoreactive deposits were mainly found aggregated in the perinuclear regions. Abundant deposits were also found dispersed throughout the mucous covering the gastric surface (Fig. 4a).

#### Adult

Gastric mucosa was covered by a thin superficial layer intensely reactive for Tβ_4_ extending to gastric foveolae. Immunoreactivity for Tβ_4_ in gastric glands was restricted to chief and oxyntic cells (Fig. 4b).

### Ileum

#### Foetus (21 weeks of gestation)

**Figure 5:**
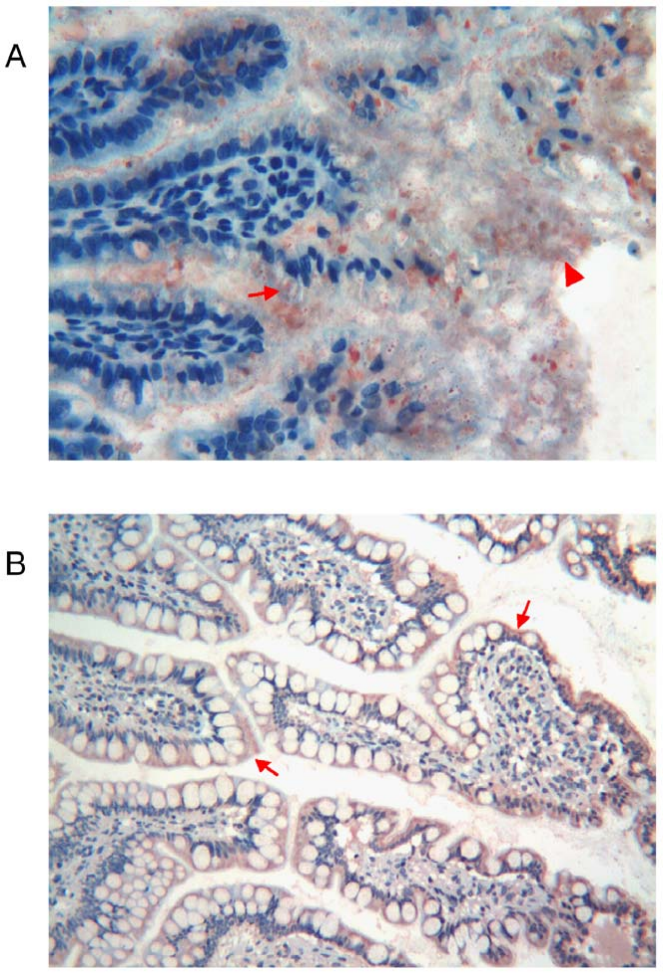
Immunohistochemical detection of thymosin β4 in foetal and adult ileum. **a**) Foetal ileum: a granular reactivity for Tβ_4_ is present in the epithelium covering ileal villi (arrow). A reactivity for the peptide is also observed in the mucous occupying the intestinal lumen (arrowhead). (Original Magnification ×400) **b**) Adult ileum: a mild reactivity for Tβ_4_ is present in the cytoplasm of enterocytes covering villi (arrows). (Original Magnification ×250)

A granular reactivity for Tβ_4_ was detected in the epithelium covering the ileal villi, more evident in the cytoplasm of mucous cells (Fig. 5a). A reactivity for the peptide was also observed in the mucous occupying the intestinal lumen.

#### Adult

A mild but diffuse reactivity for Tβ_4_ was present in the cytoplasm of enterocytes covering villi (Fig. 5b). Fine granular deposits were also observed in the cytoplasm of mucous cells.

### Colon

#### Foetus (21 weeks of gestation)

**Figure 6:**
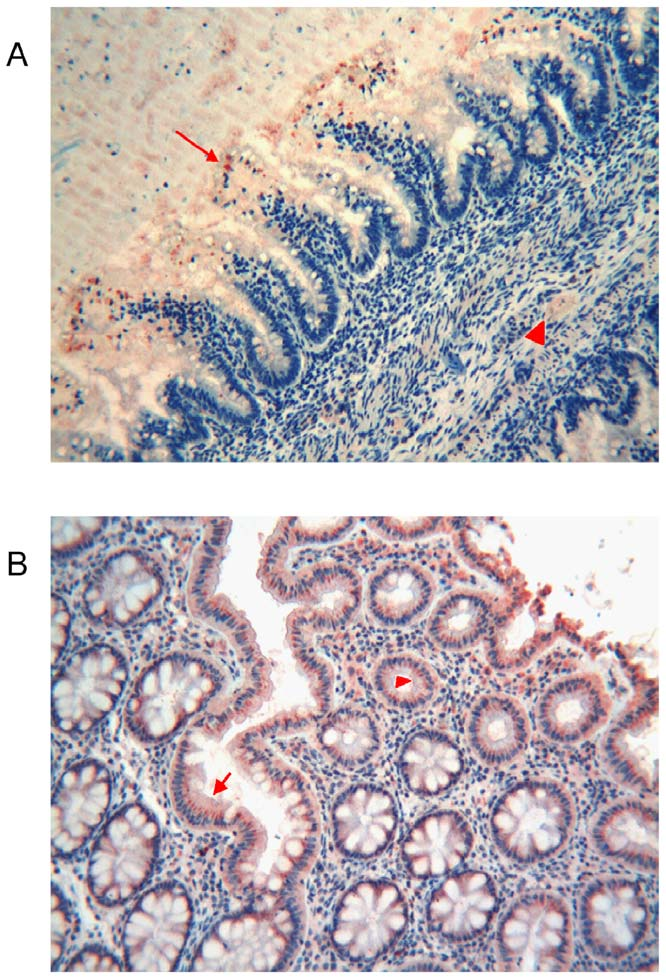
Immunohistochemical detection of thymosin β4 in foetal and adult colon. **a**) Foetal colon: coarse granules immunoreactive for Tβ_4_ are observed in the cytoplasm of superficial enterocytes (arrow). A weak positivity is detected in nervous cells (arrowhead). (Original Magnification ×250) **b**) Adult colon: an intense and diffuse reactivity for Tβ_4_, characterized by coarse sovranuclear granules is present in the superficial (arrow) and crypt epithelium (arrowhead). (Original Magnification ×250)

Coarse granules immunoreactive for Tβ_4_ were observed in the cytoplasm of superficial colon epithelial cells. No reactivity was found in the crypts (Fig. 6a). The muscular layer did not show any significant reactivity for the peptide. A weak positivity was detected in the nervous cells.

#### Adult

An intense and diffuse reactivity with coarse sovranuclear granules immunoreactive for Tβ_4_ were present in the superficial and crypt epithelium. (Fig. 6b)

### Liver

#### Foetus (21 weeks of gestation)

**Figure 7:**
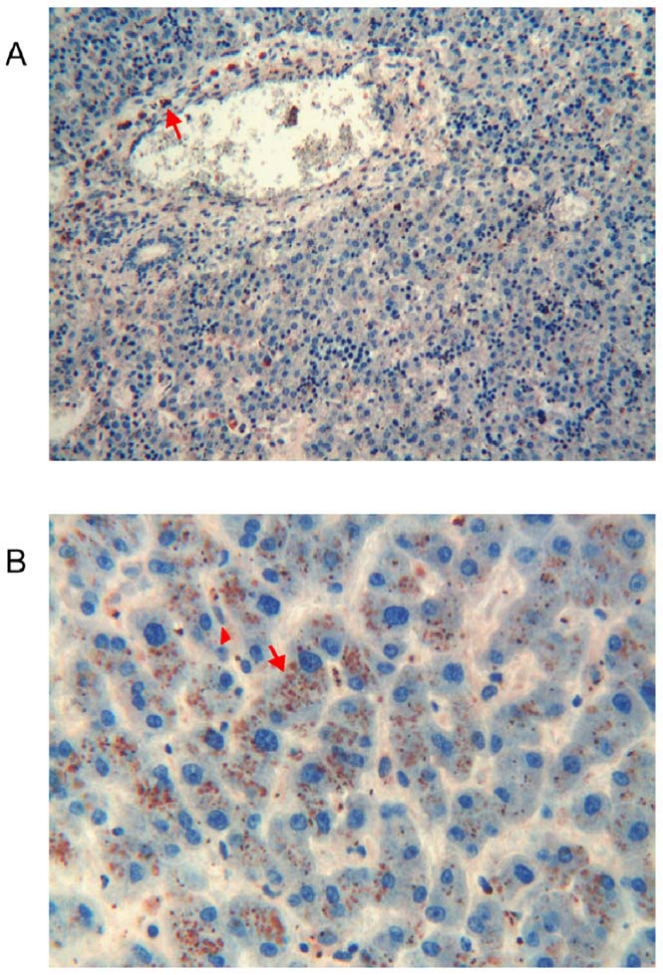
Immunohistochemical detection of thymosin β4 in foetal and adult liver. **a**) Foetal liver: no significant reactivity for Tβ_4_ is found in hepatocytes and in ductal cells. Scattered large cells in immature portal tracts show a strong immunoreactivity for the peptide (arrow). (Original Magnification ×250) **b**) Adult liver: a strong granular immunoreactivity for Tβ_4_ is found in the cytoplasm of the majority of hepatocytes in all acinar zones (arrow) and in activated Kupffer cells (arrowhead). Portal tracts, including bile ducts are negative. (Original Magnification ×400)

No significant reactivity for Tβ_4_ was found in hepatocytes and in biliary ducts nor in biliary epithelial cells of the ductal plate in the foetal liver examined in this study. A weak reactivity was frequently observed in the red blood cells in the dilated sinusoids. Only occasionally, scattered large cells in immature portal tracts showed a strong Tβ_4_ immunoreactivity (Fig. 7a).

#### Adult

The expression pattern for Tβ_4_ changed completely in immunostained adult livers. A strong granular immunoreactivity was found in the cytoplasm of the majority of hepatocytes in all acinar zones and in activated Kupffer cells (Fig. 7b). Portal tracts, including bile ducts were constantly negative.

### Pancreas

#### Foetus (20 weeks of gestation)

**Figure 8:**
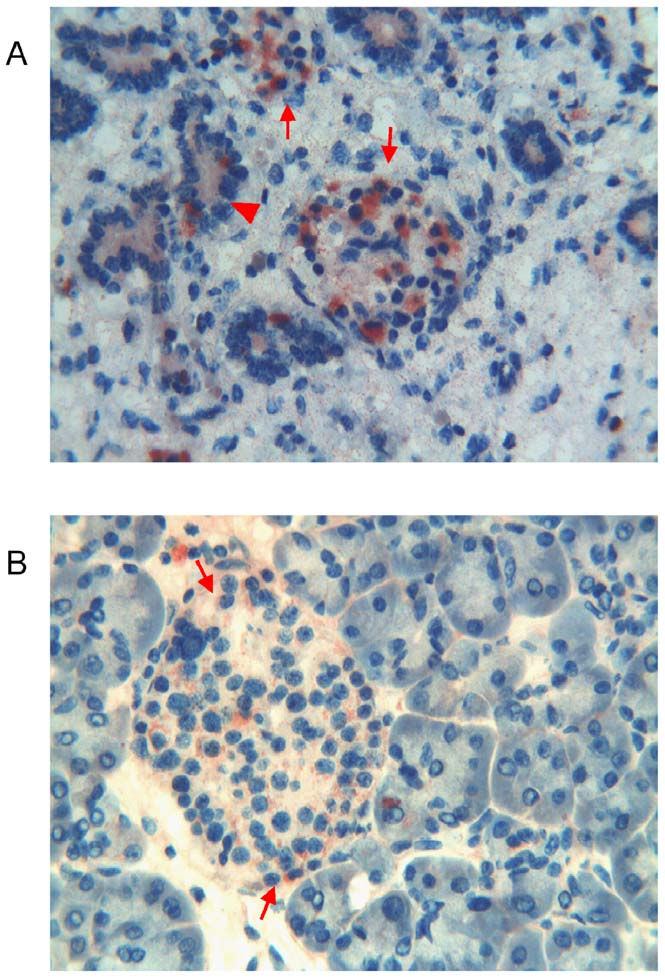
Immunohistochemical detection of thymosin β4 in foetal and adult pancreas. **a**) Foetal pancreas: immunoreactivity for Tβ_4_ is localized inside the islets of Langherans. Some of endocrine cells show the entire cytoplasm strongly immunoreactive for the peptide (arrows). Few Tβ_4_-reactive cells are also present in the exocrine pancreas, inside the tubular structures (arrowhead). (Original Magnification ×400). **b**) Adult pancreas: the highest reactivity for Tβ_4_ is observed in Langherans islet cells, which show diffuse fine granular deposits in their cytoplasm (arrows). (Original Magnification ×400).

Immunoreactivity for Tβ_4_ appeared focal, mainly localized inside the islets of Langerhans. A part of the endocrine cells showed the entire cytoplasm strongly immunoreactive for the peptide. Few Tβ_4_-reactive cells were also present in the exocrine pancreas, inside the tubular structures, intermingled among the tubular epithelium. A weak granular reactivity for the peptide was also found inside the cytoplasm of the tubular cells, a pattern suggestive for a secretion of Tβ_4_ in the pancreatic juice (Fig. 8a).

#### Adult

The distribution of Tβ_4_ immunoreactivity paralleled that observed in the foetus: the highest reactivity was observed in the islet cells which showed diffuse fine cytoplasmic granular deposits in the majority of endocrine cells. Only rare acinar cells contained cytoplasmic vacuoles reactive for Tβ_4_ (Fig. 8b).

## Discussion

Tβ_4_, the most abundant member in human cells of the thymosin family, has been initially embraced as the ideal actin monomer-sequestering peptide [18], and its function was restricted to regulate actin polymerization of non-muscle cells [19]. Further data, suggesting a role of Tβ_4_ in modulating stem cell migration [9], activation [20] and inhibition [21], as well a relation between Tβ_4_ and integrin signalling [22] induced some authors to speak about “the β-thymosin enigma” [9]. The oxidized form of Tβ_4_, thymosin beta 4 sulfoxide, has been demonstrated to have an important anti-inflammatory activity inhibiting neutrophil chemotaxis in vitro [23] and in vivo [7].

This study represents the first comprehensive analysis of Tβ_4_ immunoreactivity in the human gastrointestinal tract, pancreas and liver of human foetus in comparison to adult. Interesting differences were found between foetus and adult Tβ_4_ immunoreactivity: in some organs, such as liver, the immunoreactivity of Tβ_4_ was higher in the adult, while in others, such as endocrine pancreas, it was lower. Differences in Tβ_4_ expression between the foetus and the adult have been previously reported by our group in human salivary glands [15]. In that study developing major and minor salivary glands showed a marked reactivity for Tβ_4_, which contrasted with the low levels of immunoreactivity observed in the adult glands. In this study liver showed an opposite pattern, characterized by low reactivity for the peptide in the intrauterine life and much higher levels in the adult life. All together, these data seem to indicate the existence of an exquisite cell type- and differentiation stage-specific regulation and expression pattern of Tβ_4_ in the human gut and annexed glands during development. As a consequence, Tβ_4_ could play different roles in different organs during the organogenesis, and these roles should change during adult life.

Immunoreactivity for Tβ_4_ was detected in all different intestinal segments and in all glands examined, but with striking differences among different sites: pancreas and liver of adults showed the highest levels of reactivity for Tβ_4_, while the lowest reactivity was observed in the developing liver. A remarkable heterogeneity of Tβ_4_ immunoreactivity within the developing gastrointestinal tract was observed, ranging from diffuse immunoreactivity in pancreas and enterocytes, to peptide absence in the foetal hepatocytes. It should be outlined that some differences observed in Tβ_4_ immunoreactivity between fetuses and adult organs may be related to the different degree of cell differentiation. For example, chief and oxyntic cells, the site of Tβ_4_ storage in the adult stomach, are not well differentiated yet in the foetal stomach glands. Noticeable interindividual differences during gut development, regarding the intensity of the positivity for Tβ_4_ and its subcellular localization, were also observed (data not reported).

On the whole, the high amounts of Tβ_4_ here reported in the human gut during development are in agreement with previous experimental studies on the role of Tβ_4_ in organogenesis. High levels of Tβ_4_ mRNA had been indeed reported in early mouse postimplantation embryos [24] and in the ventricular myocardium of mice at embryonic day 10 [25]. The essential role of Tβ_4_ in heart development had been demonstrated by the generation of mice with RNAi-mediated cardiac specific knockdown of Tβ_4_.

Tβ_4_-mutant hearts showed severe impairment in the mobilization of cardiac progenitor cells, resulting in impaired cardiac development and survival [26]. Our data clearly indicate a major expression of Tβ_4_ during the development of the human gastrointestinal tract. Such a strong expression should be related to relevant roles in gut development. The well known function of Tβ_4_ in the regulation of the equilibrium betwen globular and filamentous actin [27] could partly explain the strong Tβ_4_ immunoreactivity found in the cytoplasm of enterocytes in this study. Tβ_4_ has been hypothesized to also function as a sentinel of the cell oxidative stress, its oxidation reflecting an oxidizing environment that often correlates with cell damage [7]. Lymphoid Tβ_4_, a splice variant of Tβ_4_ produced by intraepithelial lymphocytes normally present in the cytoplasm of enterocytes, has been shown to have a great capacity to reduce oxidative stress [17]. Following this hypothesis, a strong immunohistochemical reaction for Tβ_4_ could be interpreted as a cell's response to stress, also considering that antibodies utilized in this study cannot discriminate the oxidized Tβ_4_ from the non-oxidized one. Recently, Tβ_4_ has been shown to be involved in regulating phagocytosis of apoptotic cells mediated by stabilin-2 [28]. Given the fundamental role of apoptosis in embryology, our finding of a strong reactivity for Tβ_4_ in the developing gut as well as in developing salivary glands [15] reinforces the hypothesis of an important role of Tβ_4_ in organ development during embryogenesis. Future studies will be necessary to shed light on the link between Tβ_4_ and organogenesis, with particular emphasis on the role plaied by the peptide in cell response to oxidative stress, in apoptosis, in actin metabolism in the different phases of fetal, embryo and adult life. The heterogeneity of Tβ_4_ immunoreactivity detected in different organs of gastrointestinal tract and the differences observed between intrauterine and adult life should be taken into account when the role of Tβ_4_ in human physiology is assessed.
